# Training and retention effects of paced and music-synchronised walking exercises on pre-older females: an interventional study

**DOI:** 10.1186/s12877-022-03598-z

**Published:** 2022-11-23

**Authors:** Yi Wang, Xian Guo, Hongchu Wang, Yinru Chen, Naxin Xu, Minghao Xie, Duo Wai-Chi Wong, Wing-Kai Lam

**Affiliations:** 1 Department of Physical Education, Renmin University of China, Beijing, China; 2 Sports and Social Development Research Center, Renmin University of China, Beijing, China; 3 Sport Science School, Beijing Sport University, Beijing, China; 4 School of Mathematical Sciences, South China Normal University, Guangzhou, China; 5 College of Education, Beijing Sport University, Beijing, China; 6 National Institute of Sports Medicine, Beijing, China; 7 Department of Biomedical Engineering, Faculty of Engineering, The Hong Kong Polytechnic University, Hung Hom Hong Kong, China; 8 Research Institute for Sports Science and Technology, The Hong Kong Polytechnic University, Hung Hom Hong Kong, China; 9 Sports Information and External Affairs Centre, Hong Kong Sports Institute, Shatin Hong Kong, China

**Keywords:** Heart rate, Aerobic exercise, Cardio-metabolic biomarkers, Dynamic balance

## Abstract

**Background:**

Physical activity at pre-older ages (55–64 years) can greatly affect one’s physical fitness, health, physical-activity behaviour, and quality of life at older ages. The objective of this study was to conduct a 24-week walking-exercise programme among sedentary pre-older females and investigate the influence of different walking cadences on cardiorespiratory fitness and associated biomarkers.

**Methods:**

A total of 78 pre-older sedentary female participants were recruited and randomly assigned to normal (*n* = 36), paced (*n* = 15), music-synchronised (*n* = 15) walking, and no-exercise control (*n* = 12) groups, respectively. The normal, paced, and music-synchronised walking groups walked at a cadence of 120 steps/min, 125 steps/min, and 120–128 steps/min, respectively, under supervised conditions. Anthropometric characteristics, step length, nutrient intake, blood pressure and composition, and cardiorespiratory fitness were measured at baseline, the 12th week of the programme, the 24th week of the programme (completion), and after a 12-week retention period, which began immediately upon completion of the programme and did not feature any supervised exercises.

**Results:**

All walking conditions improved high-density lipoprotein cholesterol (HDL-C), low-density lipoprotein cholesterol, step length, maximum oxygen consumption (VO_2_max), and oxidative capacity at anaerobic threshold (all *P* < 0.001); however, after the 12-week retention period only the training effects of HDL-C (*P* < 0.05) and VO_2_max (*P* < 0.05) remained robust. Additionally, music-synchronised walking was found to reduce the fat ratio (*P* = 0.031), while paced walking was found to reduce body mass (*P* = 0.049).

**Conclusions:**

The significant pre–post changes in health-related outcomes across the 24-week walking intervention, including improved blood composition, longer step length, and better cardiorespiratory capacity, show that this intervention is promising for improving health and fitness. When, during the retention period, the participants resumed their usual lifestyles without supervised exercise, most physiological biomarkers deteriorated. Thus, for sedentary middle-aged females, persistent behavioural change is necessary to retain the health benefits of physical exercise.

## Background

Physical inactivity is the fourth-leading risk factor for early mortality [1]. Lack of physical activity can contribute to serious health-related problems among pre-older and older adults while, in contrast, increasing physical activity levels can lower the risk of cardiovascular morbidity and premature death [2, 3]. Pre-older adults were defined as those aged 55–65 years [4, 5]. More specifically, a recent cohort study of 204,542 individuals revealed an inverse association between physical activity and all-cause mortality for the age categories of 45–54 and 55–64 years, but no significant association for the age category of 65–74 years [6]. Meanwhile, another study has found that a metabolic equivalent of task (MET) increase in maximal aerobic capacity can reduce the risk of coronary heart disease by 15% [7].

Walking is one of the simplest and most accessible forms of exercise, and has proven health benefits for older adults. Adults are recommended to walk 10,000 steps per day to maintain good health [8]. Further, the World Health Organization recommends 150–300 minutes of moderate to high intensity or at least 75–150 minutes of vigorous-intensity physical exercise per week [9]; however, determining the optimal frequency, intensity, time, and type (known as ‘FITT’) one should perform a particular exercise can be difficult [10]. For healthy adults aged 41–85 years, a step frequency of 100–130 steps/min and 2000–9000 steps per day has been found to produce an optimal exercise level of 3–6 MET [11]. Notably, a 120–130 steps/min walking cadence, which falls within the abovementioned range, is considered practical and comfortable for people of all ages and genders [11–13]; moreover, such a cadence range accords with the American College of Sports Medicine’s (ACSM) exercise prescription guidelines [14], which advise a moderate intensity level that is between 50 and 60% maximum oxygen consumption (VO_2_max).

Paced walking (or brisk walking) represents walking at a brisk pace, usually 120 steps/min or greater, and is recognised as a suitable form of cardio workout [15]. A previous systematic review has demonstrated that a brisk-walking intervention can improve cardiorespiratory fitness, muscular strength, and body composition in older adults [10]. On the other hand, music-synchronised walking (or beat-synchronised walking) refers to walking at varied cadences in time with musical beats. Listening to music during exercise and sports has been found to have clear benefits in terms of psychophysical responses [16] and endurance performance [17, 18], and fast-paced music can stimulate breathing and heart rate during exercise. This indicates that selecting songs with progressively faster tempos may facilitate individuals’ adaptation to higher exercise intensity. Compared to paced walking (i.e. fixed, constant beats and conscious), it might be easier to maintain a proper workout intensity by walking with musical beats; this is because such an approach may help people subconsciously keep time with varying paces [13–15]. While previous studies of music-synchronised exercise have mostly focused on the intermediate effects of performance and on short-term psychophysical responses [13–15], it remains unknown whether music-synchronised walking can have positive effects on biomarkers and physiological parameters; to obtain such data, longitudinal follow-up studies are required.

Research on the physical activity of sedentary pre-older females is currently lacking, especially in regard to the interaction between music and physical activity among this population; this is particularly notable because females have been found to be more sensitive to auditory-motor coupling than males [19]. Perimenopause among women begins at 45–55 years, and the associated changes in menstrual flow and cycle length can impact physiological indicators. Furthermore, females are at a greater risk of osteoporosis than males. Walking is an appropriate physical exercise for reducing the risk of osteoporotic fractures, as it can help increase muscle strength and dynamic balance (e.g. by increasing step length) [10]. Overall, among the pre-elderly population physical activity has been found to affect physical fitness, physiological biomarkers, physical-activity behaviour, and quality of life in older age [20, 21]; therefore, understanding healthy behaviour and long-term retention effects is imperative.

Considering the above, the purpose of the present study was to investigate the effects of a 24-week walking programme on sedentary pre-older females. Four different walking cadences (no-exercise control, normal, paced, and music-synchronised) were compared, and the target variables were anthropometric measurements, step length, nutrient uptake, blood pressure and composition, and cardiorespiratory fitness. Measurements were taken before, half-way through, and after completion of the 24-week programme. Additionally, once the programme ended the participants resumed their regular behaviour (no supervised coaching), but were assessed again at 12 weeks after the programme end (retention period). We hypothesised that all walking-exercise conditions would induce significant improvement in physiological parameters, with the paced- and music-synchronised-walking conditions showing the greatest improvement after 12 weeks.

## Methods

### Participant recruitment

Using convenience sampling, a sample of healthy, sedentary, pre-older females aged between 55 and 65 years were recruited from the local community in Beijing, China. Inclusion criteria included walking fewer than 2000 steps a day and having no engagement in regular exercise over the past 3 months. To verify the number of steps the participants were taking prior to the intervention, the participants were asked to wear an Actigraph (Actigraph wGT3X+, Actigraph, Pensacola, FL, USA) on their right hip day and night (except when bathing) over seven consecutive days [22]. The Actigraph recorded vertical accelerations in magnitudes from 0.05 to 2.0 g, with a sampling rate of 30 Hz, and then summed these magnitudes over each five-second epoch. The participants had no prior experience of regularly listening to music while exercising, no history of vestibular or auditory disorders, and were confirmed to have normal hearing by an otolaryngologist. Additionally, they had not previously participated in any similar studies or exercise interventions. Participants were excluded if they were smokers or had anaemia or other cardiovascular diseases. All participants signed informed consent forms before the pre-assessment and the experiment. Ethical approval was granted by the institutional review board. The clinical trial was retrospectively registered in the Chinese Clinical Trial Registry (Reference Number: ChiCTR2200058545, Date of registration: 10/04/2022).

### Walking-exercise conditions

**Fig. 1:**
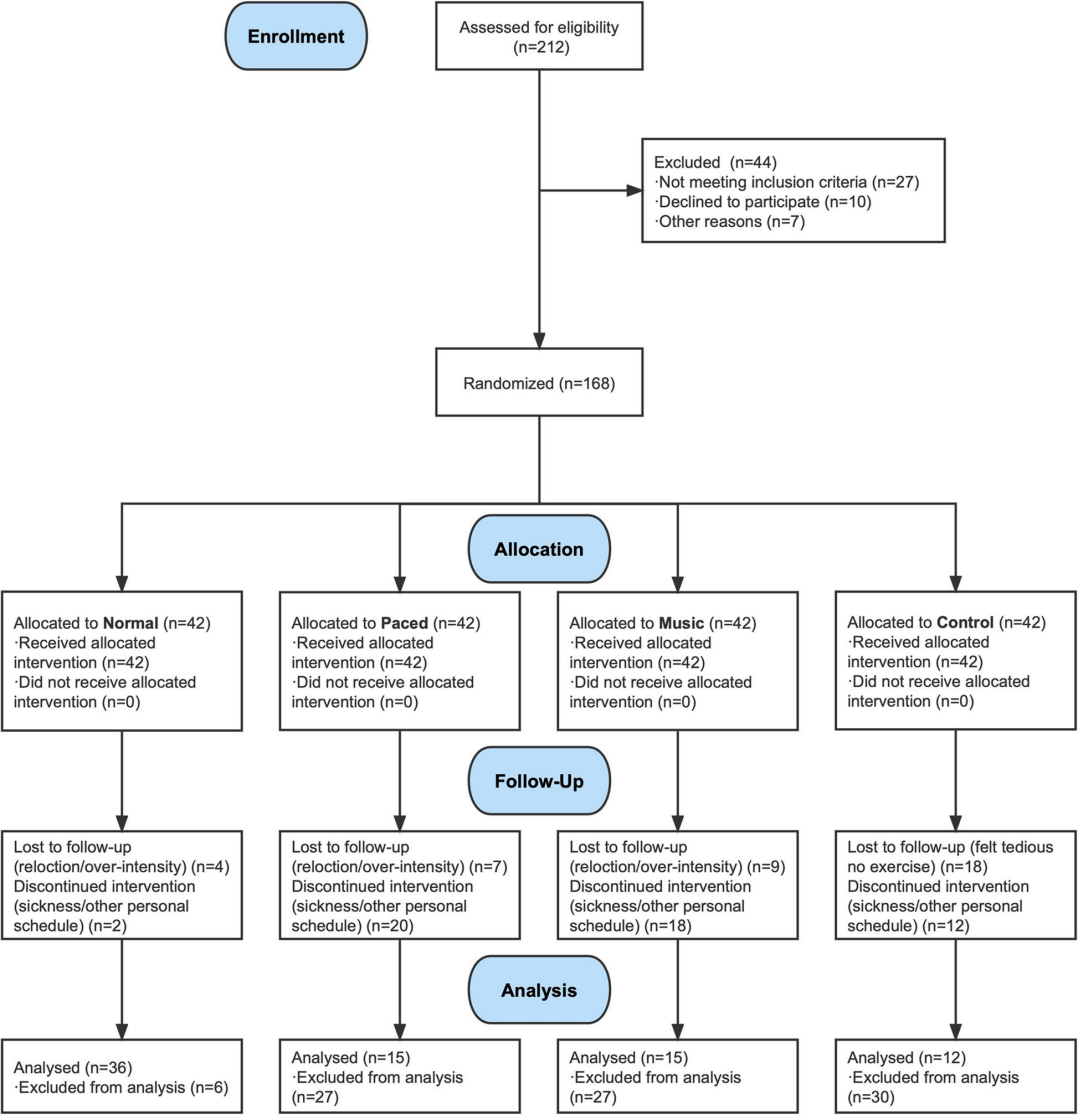
Flowchart of Participants Recruitment

To encourage completion of the 24-week exercise programme and the 12-week retention period, the participants received gain-framed monetary incentives. A total of 168 participants were enrolled and randomly assigned to four walking groups: normal walking, paced walking, music-synchronised walking, and no-exercise control. Each group contained an equal number of participants (*n* = 42), and allocation was conducted using a random number sequence generated by computer software, without allocation concealment. The flow of participant recruitment is shown in the CONSORT diagram presented in Fig. 1.

All walking groups (normal, paced, and synchronised-music walking) performed the walking exercises on the same walking pathways (located in their local residential areas) throughout the training sessions. All walking cadences were controlled by two pacemakers, one placed at the front of the walking group and one placed at the back. In the normal-walking group, the participants were instructed to walk freely for 1 hour, with an approximate step frequency of 120 steps/min. In the paced-walking group, the participants were required to walk for 1 hour with a step frequency that followed a fixed beat of 125 steps/min. The beats were counted using a metronome (Dr Beat DB 90, Boss, Japan) and played on speakers (Malone M-8622, Malone, USA) at 55 dB. The speakers were located along the walking paths to ensure that the participants could hear the beats (as specified in the pilot study). For the synchronised-music-walking group, the participants were required to walk for 1 hour with a step frequency that was in time with musical beats played on the speakers at 55 dB; this format was identical to that provided to the paced-walking group. The playlist comprised 16 music tracks that were selected based on the tracks’ ability, as determined in a previous study, to foster enjoyment and motivation to perform physical activity [23]; the tracks’ tempos varied between 120 and 128 beats per minute, which is appropriate for physical training [19, 24]. Control subjects were not assigned to any exercise programme and were asked to maintain their pre-existing physical-activity and diet behaviours.

### Preparation

Baseline physical activity level was gauged using estimated MET minutes per week (MET-min/week), as shown in Table 1. This was estimated using the validated Chinese version of the long version of the International Physical Activity Questionnaire (IPAQ) [14, 25]; using the IPAQ, the participants reported their engagement in exercise over the past 7 days, and these reports were verified using the collected actigraphy. This approach was consequently found to have excellent inter-session reliability for overall physical activity level (ICC = 0.927, 95%CI: [0.892,0.951]). All participants were required to attend a briefing session that concerned the experimental arrangement. Here, they learned how to record their nutrient intake using the Food Frequency Questionnaire (FFQ) [26, 27] and the protocol for the wearable actigraphy device. The FFQ has shown generally good (ICC = 0.54–0.68) ability to determine energy and nutrient uptake [26, 27].

**Table 1:** Demographic characteristics of the participants by groups

Variable/Group	NW (*n* = 36)	PW (*n* = 15)	MW (*n* = 15)	CTRL (*n* = 12)	*P*-value	*η*^2^
Age (year)	60.1 ± 2.7	59.0 ± 3.0	60.6 ± 3.4	60.8 ± 3.4	0.626	0.041
Height (cm)	157.9 ± 6.0	160.2 ± 6.1	159.5 ± 5.2	157.6 ± 3.5	0.898	0.035
Body mass (kg)	57.7 ± 8.1	59.0 ± 4.6	60.6 ± 5.3	59.3 ± 4.7	0.712	0.030
Lean body mass (kg)	21.8 ± 3.2	22.5 ± 2.9	21.9 ± 2.7	20.7 ± 1.1	0.634	0.039
BMI (kg/m^2^)	23.1 ± 2.6	23.0 ± 2.0	23.8 ± 1.6	23.9 ± 1.4	0.881	0.027
Physical activity level (MET-min/week)	1490.7 ± 313.8	1447.7 ± 371.2	1531.0 ± 279.7	1350.6 ± 305.5	0.113	0.032

*NW* Normal walking, *PW* Paced walking, *MW* Music-synchronized walking, *CTRL* Control

### Experimental protocol

#### Exercise procedure

All participants in the respective groups, except the no-exercise control group, performed 1-hour sessions of outdoor walking exercises; four sessions were held per week. Thus, the participants performed 4 hours of exercise per week. The entire exercise intervention comprised a total of 5760 minutes (4 h × 24 weeks = 96 h), which corresponds to the ACSM’s exercise-prescription guidelines for an intensity level equivalent to 50–60% of VO_2_max [14] (which corresponds to approximately 66–74% of maximum heart rate) [22]. The exercise sessions were supervised by two instructors, who also served as pacemakers for the lowest (i.e. the pacemaker at the back of the group) and highest (i.e. the pacemaker at the front of the group) walking speeds for each of the walking exercise groups; this was to ensure that all participants walked with the same intensity throughout the 24-week experiment. The supervisors/instructors observed, encouraged, and guided the groups in regard to walking speed and method.

The walking route was pre-determined and was located close to the participants’ residential areas. Each exercise session commenced at 6:00 pm, beginning with a 20-min warm-up. The baseline cadence was 120–130 steps/min [12, 13], adjusted to accord with the walking condition in question. For safety purposes, the wearable heart rate monitor would alarm when the heart rate exceeded 64–76% of the maximum heart rate (HRmax), which was calculated using a formula that featured age as the independent variable [14, 28]. At each walking session (outdoor), participants first performed a warm-up and activated a heart rate monitor alarm to control their heart rate at between 66 and 74% HRmax (i.e. 110–130 b/min) during the walking sessions [22]. Haptic vibration feedback was sent to the participants to help them maintain their walking intensity.

Once the 24-week exercise course had finished the retention period began (spanning from December to February; 12 weeks), during which the participants were no longer provided with an exercise schedule. During this retention period, all participants were asked to resume their usual/preferred amount of daily exercise. Weekly phone calls were made to remind the participants not to alter their physical activity or dietary behaviours.

#### Measurement and assessment

Measurements and assessments were taken at four time points (T0, T12, T24, and R12): These corresponded to the baseline (just before the start of the exercise programme; T0), halfway through the programme (T12), upon completion of the programme (i.e. at the time of programme completion; T24), and after the retention period (i.e. during the 12th week after the programme; R12). At each time point, the participants were invited to the laboratory to complete the FFQ and IPAQ; undergo anthropometric, physical-activity, and physiological measurement; and undergo a cardiorespiratory fitness test, gait analysis, and blood test. The participants were asked to avoid taking any medicine or alcohol or performing any vigorous physical activity in the 12 h preceding the measurements and assessments. Additionally, each participant was asked to fast in the morning and to empty his/her bladder and intestines before the assessments to avoid the influence of food and drink intake [29]. They were also asked to wear minimal clothing during the dual-energy X-ray absorptiometry (DXA) test; two full-body DXA scans were performed (in supine and prone positions, respectively) to analyse fat tissue and lean mass [29, 30]. Overall energy expenditure during physical activity was determined using the established IPAQ formula (i.e. cardiorespiratory endurance of health-related physical-fitness testing and analysis) [14].

Cardiorespiratory fitness tests were performed using graded treadmill walking exercises, which were conducted in accordance with the Bruce protocol [31]. Using a metabolic measurement device, the tests assessed VO_2_max at 30-second intervals and maximum aerobic speed (V_max_) (Cortex Metalyzer-III, Germany); peak heart rate (HR) and blood pressure were also measured. The participants were asked to indicate their rate of perceived exertion (RPE) on the Borg scale when they reached 50–60% of VO_2_max and when they came close to their VO_2_max. The 50–60% level of VO_2_max is considered a moderate level of physical-activity intensity, and is recommended for those who do not have a regular exercise habit [14].

Resting HR and blood pressure, including systolic blood pressure and diastolic blood pressure, were measured using a chest-belt monitor (Polar 5 pulse, Polar, Kempele, Finland) and an electronic sphygmomanometer (SunTec Tango+, SunTec, USA), respectively. Before each measurement, the participants were asked to sit quietly for 10 min. Subsequently, the participants lay in the supine position for 5 minutes. Cardiac variables, including cardiac output (CO), stroke volume (SV), and ejection fraction (EF), were measured using ultrasonography (GE Vivid 7, Diagnostx, LLC, USA). In the supine position, a power recumbent exercise bike (Angio Imaging 967,930, Lode, Netherlands) was used at a speed of 60 L/min to monitor cardiac responses (based on the VO_2_max-loaded exercise tests). Relative CO, SV, and EF were measured under loaded conditions (50% VO_2_max) and subtracted from the baseline. Blood samples (5 mL) were taken from the participants between 7:00 am and 8:00 am, after overnight fasting; all samples were obtained through venepuncture, which was performed by registered nurses. Through these samples, metabolic indicators, including triglycerides, haemoglobin A1c (HbA1c), high-density lipoprotein (HDL-C), low-density lipoprotein (LDL-C), and fasting blood glucose, which are commonly used to assess health status, were measured.

To measure step length, the participants were asked to perform walking trials at their own preferred speed in the gait laboratory. Step length was determined using 10 consecutive pressure plates (Zebris Medical, Weitnau, Germany) placed along a 20-m walkway. The pressure plates had a sampling frequency of 100 Hz and resolution of one sensor per cm^2^. Step length was determined by averaging the step lengths produced in three successive trials.

### Data analysis

All statistical analyses were performed using R statistical package (R Foundation, Vienna, Austria) and with a significance level of 0.05. The data normality of each variable for each group was checked using the Kolmogorov-Smirnov test, while Cook’s distance method was used to check for significant outliers. Significant differences between the groups in terms of baseline characteristics were checked using one-way analyses of variance (ANOVAs). The Alexander-Govern adjustment was applied if the assumption of variance homogeneity was violated.

Two-way mixed design ANOVAs (four walking groups × four time points) were conducted to evaluate the interaction effect and main effects of walking and time conditions. The assumption of sphericity was checked using Mauchly’s test, and the Greenhouse–Geisser test was used to adjust the data when sphericity was violated. If a statistically significant two-way interaction was found, a simple main effect analysis was performed. All significance levels were set at *P* < 0.05.

## Results

### Participant information

Overall, 168 participants enrolled in the experiment, of whom a total of 78 completed the programme and the follow-up retention period; this group of 78 comprised 12 from the control group, 36 from the normal group, 15 from the paced-walking group, and 15 from the music-synchronised group. Reason for participant withdrawal included sickness (*n* = 7), moving residence (*n* = 3), intolerance of the exercise intensity (*n* = 8), the tedium of being unable to perform exercise (control-group participants; *n* = 18), and inability to fit the required walking into one’s schedule (*n* = 30; Fig. 1). There were no significant differences between the exercise groups in terms of age, height, body mass/lean body mass, body mass index, or self-reported physical-activity level at baseline (*P* > 0.05, Table 1).

### Anthropometric characteristics and step length

**Fig. 2:**
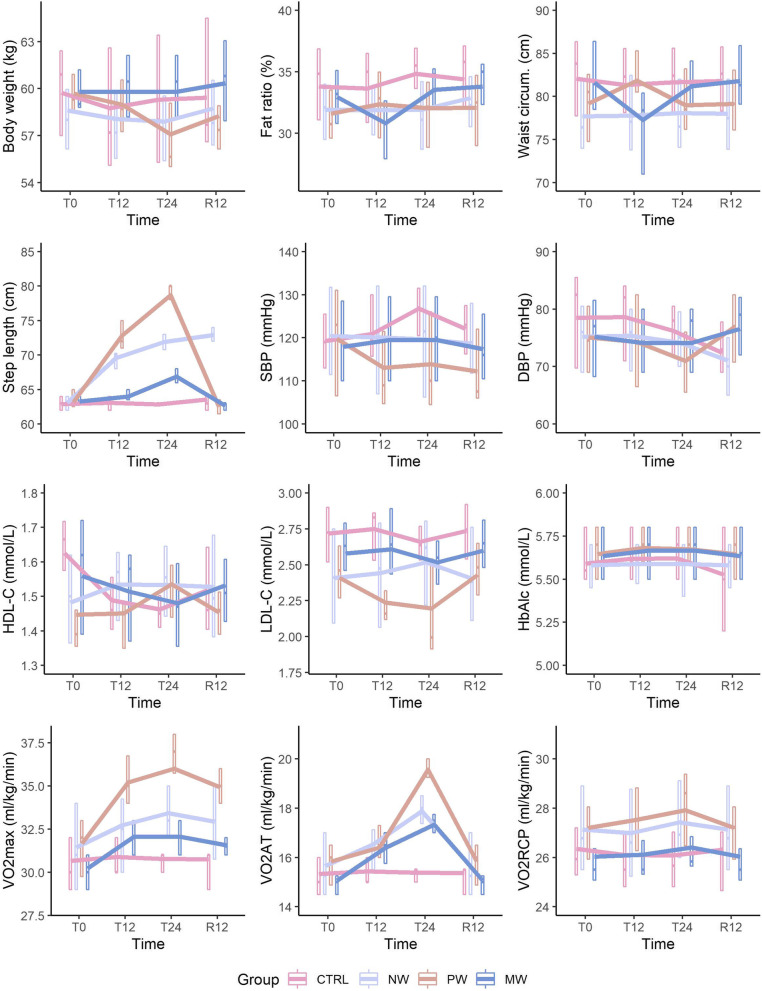
Test Variables across Time (1st Row: Body Weight, Fat Ratio, Waist Circumference; 2nd Row: Step Length, SBP, DBP; 3rd row: HDL-C, LDL-C, HbAlc; 4th row: VO2max, VO2AT, VO2RCP)

As shown in Table 2 and Fig. 2, there were significant interactions between group and time for body mass (*P* < 0.001), fat ratio (*P* < 0.001), waist circumference (*P* = 0.011), and step length (*P* < 0.001). Music-synchronised walking significantly reduced fat ratio at T12 (95%CI [− 5.58, − 0.20], *P* = 0.03) but this effect showed significant deterioration at T24 (95%CI [0.76, 6.14], *P* = 0.007) and R12 (95%CI [1.05, 6.43], *P* = 0.003). Paced walking significantly reduced body weight at T24 (95%CI [− 1.80, − 0.84], *P* < 0.001); although this effect on body weight had significantly deteriorated by R12 (95%CI [0.25, 1.21], *P* < 0.001), it remained significantly lower than that at baseline (95%CI [− 1.07, − 0.11], *P* < 0.001). In addition, all walking conditions significantly increased step length (music-synchronised: 95%CI [2.50, 4.70], paced walking: 95%CI [14.20, 19.53], normal walking: 95%CI [7.75, 8.86], *P* < 0.001), but only the normal-walking group showed no significant deterioration in step length at R12 (*P* > 0.05).

**Table 2:** Anthropometric characteristics, step length, physical activity, nutrients uptakes, and cardiorespiratory fitness variables among walking and control groups at baseline (T0), 12-week (T12), 24-week (T24) and 12-week retention (R12)

Variable/Time	Group	T0	T12	T24	R12	Effect (*P*-value, Eta Square value, [post-hoc results])		
						Interaction	Group	Time
Anthropometric characteristics and step length								
Body mass (kg)	NW	57.7 ± 8.1	57.0 ± 8.0	56.3 ± 7.4	57.1 ± 7.2	**< 0.001** *η*^*2*^ = 0.009	0.204 *η*^*2*^ = 0.058	**0.010** *η*^*2*^ = 0.006 [N.A.]
	PW	59.0 ± 4.6	57.8 ± 4.5	55.2 ± 3.8	57.1 ± 3.8			
	MW	60.6 ± 5.3	60.1 ± 4.7	60.1 ± 4.7	61.2 ± 4.7			
	CTRL	59.3 ± 4.7	59.5 ± 4.9	60.1 ± 5.2	61.5 ± 5.3			
Lean mass (kg)	NW	21.8 ± 3.2	21.9 ± 3.3	21.8 ± 3.2	21.8 ± 3.2	0.095 *η*^*2*^ = 0.012	0.193 *η*^*2*^ = 0.050	0.337 *η*^*2*^ = 0.003
	PW	22.5 ± 2.9	21.9 ± 2.7	23.7 ± 3.1	23.6 ± 3.0			
	MW	21.9 ± 2.7	22.0 ± 3.4	22.3 ± 2.3	21.9 ± 2.2			
	CTRL	20.7 ± 1.1	20.8 ± 1.2	20.8 ± 1.2	20.8 ± 1.1			
Fat ratio (%)	NW	31.2 ± 4.1	31.0 ± 4.1	31.2 ± 4.1	32.9 ± 4.2	**< 0.001** *η*^*2*^ = 0.024	0.102 *η*^*2*^ = 0.064	**< 0.001** *η*^*2*^ = 0.018 [N.A.]
	PW	31.0 ± 4.2	31.8 ± 3.8	30.6 ± 4.4	31.1 ± 4.5			
	MW	33.0 ± 3.1	30.1 ± 5.0	33.5 ± 3.1	33.8 ± 3.1			
	CTRL	33.8 ± 4.0	34.2 ± 4.3	34.5 ± 4.8	34.9 ± 4.8			
Waist circumference (cm)	NW	78.9 ± 8.5	78.6 ± 8.3	78.5 ± 8.4	78.9 ± 8.4	0.011 *η*^*2*^ = 0.016	0.818 *η*^*2*^ = 0.011	0.589 *η*^*2*^ = 0.001
	PW	76.2 ± 7.0	80.1 ± 5.9	77.6 ± 6.1	77.7 ± 6.1			
	MW	79.6 ± 7.2	76.1 ± 6.3	79.5 ± 6.2	80.0 ± 6.7			
	CTRL	80.8 ± 7.0	80.1 ± 6.6	80.5 ± 6.5	80.6 ± 6.5			
Hip circumference (cm)	NW	95.8 ± 4.3	93.9 ± 5.1	93.9 ± 4.9	94.5 ± 5.1	0.058 *η*^*2*^ = 0.012	0.564 *η*^*2*^ = 0.023	0.589 *η*^*2*^ = 0.001
	PW	93.2 ± 3.4	95.3 ± 4.8	94.1 ± 5.1	94.3 ± 4.8			
	MW	95.8 ± 4.3	93.2 ± 5.1	95.2 ± 3.8	94.6 ± 4.4			
	CTRL	95.9 ± 5.7	96.0 ± 5.6	96.2 ± 5.5	96.5 ± 5.6			
Step length (m/step)	NW	0.4 ± 0.01	0.5 ± 0.01	0.5 ± 0.02	0.5 ± 0.02	**< 0.001** *η*^*2*^ = 0.327	**< 0.001** *η*^*2*^ = 0.266 [N.A.]	**< 0.001** *η*^*2*^ = 0.288 [N.A.]
	PW	0.4 ± 0.02	0.5 ± 0.02	0.6 ± 0.02	0.4 ± 0.02			
	MW	0.4 ± 0.01	0.4 ± 0.01	0.4 ± 0.01	0.4 ± 0.01			
	CTRL	0.4 ± 0.01	0.4 ± 0.01	0.4 ± 0.01	0.4 ± 0.03			
Physical activity and nutrient uptake								
Physical activity level (MET-min/week)	NW	1490.7 ± 313.8	1425.3 ± 314.3	1452.7 ± 347.4	1484.3 ± 311.8	0.710 *η*^*2*^ = 0.020	0.89 *η*^*2*^ = 0.002	0.98 *η*^*2*^ = 0.001
	PW	1447.7 ± 371.2	1540.5 ± 277.8	1545.0 ± 278.4	1405.8 ± 327.4			
	MW	1531.0 ± 279.7	1513.6 ± 242.2	1492.1 ± 238.3	1443.7 ± 338.2			
	CTRL	1350.6 ± 305.5	1437.0 ± 411.4	1490.8 ± 378.1	1544.1 ± 312.7			
Dietary intake (kcal)	NW	2702.7 ± 1.5	2727.9 ± 1.5	2759.9 ± 1.5	2393.9 ± 1.5	0.150 *η*^*2*^ = 0.016	0.514 *η*^*2*^ = 0.022	0.250 *η*^*2*^ = 0.005
	PW	2702.8 ± 1.5	2727.9 ± 1.5	2759.9 ± 1.5	2394.0 ± 1.5			
	MW	2703.1 ± 1.5	2728.1 ± 1.5	2760.1 ± 1.5	2393.7 ± 1.5			
	CTRL	2703.2 ± 1.5	2727.7 ± 1.5	2759.7 ± 1.5	2394.0 ± 1.4			
Carbohydrate intake (g/day)	NW	360.2 ± 8.0	363.8 ± 8.0	365.6 ± 8.0	353.5 ± 8.0	0.843 *η*^*2*^ = 0.001	0.816 *η*^*2*^ = 0.012	0.496 *η*^*2*^ = 0.001
	PW	368.4 ± 4.1	372.0 ± 4.1	373.8 ± 4.1	361.7 ± 4.1			
	MW	360.5 ± 7.6	364.1 ± 7.6	365.9 ± 7.6	353.8 ± 7.6			
	CTRL	358.6 ± 7.5	362.3 ± 7.4	364.1 ± 7.4	352.0 ± 7.4			
Fat intake (g/day)	NW	95.8 ± 2.3	92.1 ± 3.8	92.9 ± 3.8	67.7 ± 3.8	0.240 *η*^*2*^ = 0.027	0.336 *η*^*2*^ = 0.020	0.225 *η*^*2*^ = 0.010
	PW	96.4 ± 1.5	95.7 ± 1.6	96.5 ± 1.6	71.3 ± 1.6			
	MW	95.6 ± 1.6	94.6 ± 1.6	95.4 ± 1.6	70.2 ± 1.6			
	CTRL	94.7 ± 1.3	89.8 ± 2.6	90.6 ± 2.6	65.4 ± 2.6			
Protein intake (g/day)	NW	90.8 ± 1.8	90.3 ± 1.4	95.6 ± 2.2	76.0 ± 2.2	0.441 *η*^*2*^ = 0.017	0.401 *η*^*2*^ = 0.021	0.964 *η*^*2*^ = 0.0002
	PW	91.2 ± 1.3	94.0 ± 1.5	97.7 ± 1.1	78.1 ± 1.1			
	MW	90.8 ± 1.4	93.7 ± 1.3	95.8 ± 1.5	76.2 ± 1.5			
	CTRL	90.2 ± 1.4	91.5 ± 1.3	95.7 ± 1.6	76.1 ± 1.6			
Blood pressure and composition								
Systolic blood pressure (SBP, mmHg)	NW	120.4 ± 15.5	120.8 ± 14.5	119.6 ± 12.6	145.2 ± 22.0	0.036 *η*^*2*^ = 0.009	0.040 *η*^*2*^ = 0.093	**< 0.001** *η*^*2*^ = 0.015 [R12 > T0,T12,T24]
	PW	122.1 ± 17.3	116.8 ± 16.2	113.9 ± 11.0	138.7 ± 20.8			
	MW	122.1 ± 16.1	119.5 ± 12.0	119.5 ± 12.0	147.0 ± 12.0			
	CTRL	132.5 ± 18.4	131.1 ± 17.1	130.8 ± 11.9	152.1 ± 22.3			
Diastolic blood pressure (DBP, mmHg)	NW	74.6 ± 8.5	75.2 ± 8.4	73.6 ± 6.8	68.9 ± 9.4	**0.036** *η*^*2*^ = 0.038	0.358 *η*^*2*^ = 0.026	0.077 *η*^*2*^ = 0.014
	PW	76.1 ± 11.2	74.3 ± 9.1	71.0 ± 6.1	75.6 ± 13.0			
	MW	76.3 ± 8.9	74.1 ± 6.4	74.1 ± 6.4	77.9 ± 8.7			
	CTRL	77.8 ± 10.5	76.9 ± 9.4	76.3 ± 7.3	76.6 ± 11.5			
Triglycerides (TG, mmol/L)	NW	1.5 ± 0.7	1.5 ± 0.7	1.4 ± 0.4	1.2 ± 0.6	0.388 *η*^*2*^ = 0.008	0.975 *η*^*2*^ = 0.002	**< 0.001** *η*^*2*^ = 0.047 [T0,T12,T24 > R12]
	PW	1.5 ± 0.4	1.3 ± 0.3	1.2 ± 0.3	1.3 ± 0.4			
	MW	1.5 ± 0.6	1.4 ± 0.5	1.4 ± 0.5	1.1 ± 0.3			
	CTRL	1.5 ± 0.5	1.5 ± 0.5	1.4 ± 0.3	1.2 ± 0.5			
High-density lipoprotein cholesterol (HDL-C, mmol/L)	NW	1.4 ± 0.3	1.5 ± 0.4	1.5 ± 0.3	1.6 ± 0.3	**< 0.001** *η*^*2*^ = 0.029	0.860 *η*^*2*^ = 0.009	**< 0.001** *η*^*2*^ = 0.028 [N.A.]
	PW	1.4 ± 0.3	1.5 ± 0.4	1.7 ± 0.3	1.5 ± 0.3			
	MW	1.5 ± 0.3	1.4 ± 0.3	1.4 ± 0.3	1.6 ± 0.3			
	CTRL	1.5 ± 0.3	1.3 ± 0.2	1.4 ± 0.2	1.6 ± 0.3			
Low-density lipoprotein cholesterol (LDL-C, mmol/L)	NW	2.6 ± 0.5	2.5 ± 0.5	2.4 ± 0.5	2.6 ± 0.5	**< 0.001** *η*^*2*^ = 0.017	0.268 *η*^*2*^ = 0.046	< **0.001** *η*^*2*^ = 0.034 [N.A.]
	PW	2.6 ± 0.7	2.4 ± 0.6	2.0 ± 0.5	2.6 ± 0.7			
	MW	2.6 ± 0.7	2.6 ± 0.6	2.5 ± 0.5	2.7 ± 0.7			
	CTRL	2.8 ± 0.6	2.7 ± 0.5	2.8 ± 0.5	2.9 ± 0.6			
HbA1c (mmol/L)	NW	5.7 ± 0.3	5.6 ± 0.3	5.6 ± 0.3	5.7 ± 0.3	**0.001** *η*^*2*^ = 0.007	0.487 *η*^*2*^ = 0.030	0.233 *η*^*2*^ = 0.001
	PW	5.7 ± 0.4	5.8 ± 0.4	5.7 ± 0.4	5.7 ± 0.4			
	MW	5.8 ± 0.3	5.8 ± 0.3	5.8 ± 0.3	5.8 ± 0.3			
	CTRL	5.7 ± 0.3	5.7 ± 0.3	5.7 ± 0.3	5.5 ± 0.3			
Fasting blood glucose (FBG, mmol/L)	NW	5.2 ± 0.4	5.6 ± 1.5	5.3 ± 0.3	5.2 ± 0.4	0.611 *η*^*2*^ = 0.016	0.325 *η*^*2*^ = 0.018	0.286 *η*^*2*^ = 0.010
	PW	5.4 ± 0.6	5.4 ± 0.2	5.3 ± 0.4	5.4 ± 0.6			
	MW	5.5 ± 0.6	5.4 ± 0.4	5.4 ± 0.4	5.5 ± 0.6			
	CTRL	5.5 ± 0.5	5.6 ± 0.5	5.6 ± 0.4	5.6 ± 0.5			
Cardiorespiratory fitness								
Relative cardiac output (CO, L/min)	NW	0.8 ± 0.1	0.7 ± 0.3	0.5 ± 0.7	1.0 ± 0.1	0.928 *η*^*2*^ = 0.001	0.349 *η*^*2*^ = 0.038	**< 0.001** *η*^*2*^ = 0.032 [T24 > R12 > T12 > T0]
	PW	0.6 ± 1.3	0.4 ± 1.3	0.4 ± 1.3	0.8 ± 1.3			
	MW	1.1 ± 1.1	1.0 ± 1.1	0.9 ± 1.1	1.2 ± 1.1			
	CTRL	0.8 ± 0.2	0.6 ± 0.1	0.4 ± 0.1	0.8 ± 0.6			
Relative stroke volume (SV, ml)	NW	3.8 ± 9.5	5.0 ± 8.6	8.2 ± 10.6	3.3 ± 10.6	0.528 *η*^*2*^ = 0.008	0.840 *η*^*2*^ = 0.008	0.005 *η*^*2*^ = 0.019
	PW	5.6 ± 12.8	7.2 ± 8.9	7.21 ± 1.1	5.5 ± 9.6			
	MW	5.7 ± 12.6	6.6 ± 11.4	11.5 ± 10.2	6.5 ± 12.6			
	CTRL	6.3 ± 10.8	5.1 ± 9.9	6.0 ± 5.1	5.8 ± 11.1			
Relative ejection fraction (EF, %)	NW	6.3 ± 9.5	6.8 ± 9.2	6.0 ± 4.9	6.2 ± 9.5	0.275 *η*^*2*^ = 0.014	0.578 *η*^*2*^ = 0.019	0.276 *η*^*2*^ = 0.005
	PW	9.4 ± 12.7	5.1 ± 11.8	6.4 ± 7.9	9.3 ± 12.7			
	MW	8.7 ± 5.7	9.5 ± 5.1	5.0 ± 7.2	8.7 ± 5.7			
	CTRL	3.7 ± 10.8	6.0 ± 10.5	3.6 ± 1.6	3.7 ± 10.2			
Maximal oxygen uptake (VO_2_max, ml/kg/min)	NW	30.5 ± 3.7	32.2 ± 3.8	34.1 ± 2.5	32.4 ± 3.8	**0.001** *η*^*2*^ = 0.048	**< 0.001** *η*^*2*^ = 0.172 [N.A.]	**< 0.001** *η*^*2*^ = 0.110 [N.A.]
	PW	30.1 ± 4.0	35.5 ± 2.4	36.7 ± 2.8	35.3 ± 2.6			
	MW	29.3 ± 3.0	32.1 ± 2.1	32.1 ± 2.1	31.1 ± 2.6			
	CTRL	29.3 ± 3.1	29.7 ± 2.6	29.3 ± 3.1	28.8 ± 3.4			
Oxidative capacity at anaerobic threshold (VO_2_AT, ml/kg/min)	NW	15.4 ± 1.5	16.5 ± 1.3	17.9 ± 0.9	15.4 ± 1.5	**< 0.001** *η*^*2*^ = 0.084	**< 0.001** *η*^*2*^ = 0.113 [N.A.]	**< 0.001** *η*^*2*^ = 0.297 [N.A.]
	PW	15.0 ± 2.0	16.4 ± 1.2	19.6 ± 0.7	15.0 ± 2.0			
	MW	15.1 ± 0.9	16.4 ± 0.8	17.3 ± 0.8	15.1 ± 0.9			
	CTRL	14.7 ± 1.5	14.8 ± 1.3	14.7 ± 1.5	14.4 ± 1.7			
Oxidative capacity at respiratory compensation point (VO2RCP, ml/kg/min)	NW	25.9 ± 3.1	26.3 ± 2.8	26.7 ± 2.7	25.9 ± 3.1	**< 0.001** *η*^*2*^ = 0.007	0.376 *η*^*2*^ = 0.039	**< 0.001** *η*^*2*^ = 0.018 [N.A.]
	PW	25.6 ± 3.4	26.5 ± 2.7	27.5 ± 2.4	25.6 ± 3.4			
	MW	24.9 ± 2.5	25.4 ± 2.4	25.7 ± 2.3	24.9 ± 2.5			
	CTRL	24.9 ± 2.6	25.0 ± 2.5	24.9 ± 2.5	24.7 ± 2.8			

*NW* Normal walking, *PW* Paced walking, *MW* Music-synchronized walking, *CTRL* Control, *N.A.* Not applicable to report the post-hoc results due to the present of the interaction effect

### Physical-activity level and nutrient uptakes

As shown in Table 2, there were no significant interactions between groups and time (*P* > 0.05). Moreover, the main effects of group and time point were also insignificant (*P* > 0.05). In other words, walking condition did not induce significant changes in physical activity or nutrient uptake levels.

### Blood pressure and blood composition

As shown in Table 2 and Fig. 2, there were significant interactions between systolic pressure (*P* = 0.036), diastolic pressure (*P* = 0.036), HDL-C (*P* < 0.001), LDL-C (*P* < 0.001), and HbA1c (*P* = 0.001). The normal-walking group showed lower diastolic pressure at R12 than at other timepoints (T0–R12: 95%CI [2.43, 9.07], T12–R12: 95%CI [3.02, 9.65], T24–R12: 95%CI [1.46, 8.10], *P* < 0.001). Meanwhile, although the paced-walking group showed significantly increased HDL-C levels during the programme, their HDL-C levels showed significant deterioration at R12 (95%CI [− 0.28, − 0.01], *P* = 0.03). The music-synchronised-walking and normal-walking groups showed the highest HDL-C level at R12 (music-synchronised: R12–T0: 95%CI [0.06, 0.20], R12–T12: 95%CI [0.08, 0.22], R12–T24: 95%CI [0.07, 0.21]; normal walking: R12–T0 95%CI [0.06, 0.20], R12–T12: 95%CI [0.01, 0.16], R12–T24 95%CI [− 0.06, 0.09], *P* < 0.05). All walking groups showed significantly reduced LDL-C at T24 (music-synchronised: 95%CI [− 0.29, − 0.07], paced walking: 95%CI [− 0.82, − 0.39], normal walking: 95%CI [− 0.29, − 0.06], *P* < 0.05) but, for each group, this effect significantly deteriorated by R12 (music-synchronised: 95%CI [0.09, 0.31], paced walking: 95%CI [0.41, 0.84], normal walking: 95%CI [0.09, 0.31], *P* < 0.05).

### Cardiorespiratory fitness

As shown in Table 2 and Fig. 2, there were significant interactions among VO_2_max (*P* = 0.001), oxidative capacity at anaerobic threshold (VO_2_AT; *P* < 0.001), and at the respiratory compensation point (*P* < 0.001). All walking groups showed significantly increased VO_2_max (music-synchronised: 95%CI [1.01, 4.46], paced walking: 95%CI [4.16, 9.18], normal walking: 95%CI [1.74, 5.37], *P* < 0.05) and VO_2_AT (music-synchronised: 95%CI [1.59, 2.94], paced walking: 95%CI [3.53, 5.53], normal walking: 95%CI [1.74, 3.11], *P* < 0.05). However, for all groups VO_2_AT significantly deteriorated at R12 (music-synchronised: 95%CI [− 2.01, − 0.66]; paced walking: 95%CI [− 5.53, − 3.53]; normal walking: 95% CI [− 3.11, − 1.74], *P* < 0.05).

## Discussion

This study examined the effects of different walking exercises on various physiological parameters. A follow-up assessment was conducted 12 weeks post-intervention (during which the participants resumed their regular/preferred daily behaviours) to evaluate the retention effects of the exercises. The novelty of this study concerns its evaluation of the physiological improvements induced by music-synchronised exercise and its documentation of the retention of such effects upon resumption of their usual lifestyle. The outcomes of this study can provide evidence for exercise protocol design and directions for health promotion.

Our analysis showed that physical-activity level and nutrient intake did not change significantly throughout the study period. This finding confirms that these confounding factors were appropriately controlled for. All walking groups (normal, paced, and synchronised music) appeared to experience some positive outcomes in relation to body anthropometry, step length, blood pressure, biomarkers, and cardiorespiratory fitness. Step length determines individuals’ walking-function capacity [32, 33] and, after the intervention (T24), all walking groups showed longer step lengths than the control group. Step length decreases with age [34], and increasing one’s step length may lead to improvements in lower-limb muscle strength and balance ability [35]. Walking function can be further confirmed by assessing gait asymmetry and variability after prolonged treadmill walking [36, 37].

Variables such as body mass, fat ratio, and waist circumference are predictors of cardiorespiratory diseases [38]. Our sample showed an improvement in cardiorespiratory fitness parameters, which may indicate an improvement in their general health. More specifically, the three walking groups showed improvements in VO_2_max and VO_2_AT (despite deterioration in VO_2_AT at follow-up). This suggests that walking can improve the anaerobic capacity efficiency of individuals with sedentary lifestyles, thereby elevating their health level. Additionally, the walking groups’ relative CO and SV (i.e. loaded values subtracted from baseline values) had improved by programme completion (T24), which indicated a higher efficiency of cardiac function and, thus, high efficiency regarding the delivery of oxygen, nutrients, and chemicals to body cells and the removal of metabolic waste. Relative SV denotes cardiac function, while relative CO denotes the total volume output of blood (i.e. heart rate × relative stroke volume). Improved cardiac function can enhance anaerobic efficiency [39].

All walking conditions also showed increased HDL-C and reduced LDL-C levels by T24, but the effect on LDL-C levels had deteriorated by follow-up. It should be noted, however, that blood composition variables require a relatively long time to respond and adapt to changes in exercise frequency. Such improvements in blood composition variables provide supporting evidence that controlled fast-paced walking is superior for promoting muscle elasticity during walking. In our study, both paced and music-synchronised walking were controlled using auditory tones; musical beats are considered to induce cohesion between the central nervous system and fatigue perception, which may distract attention from levels of fatigue and encourage increased muscle power output during physical exercise [40, 41]. However, in our study the music-synchronised group did not show similar benefits to the paced-walking group. This may have been due to the fact that the music-synchronised group was exposed to a range of beats across different song tracks (fast, slow, slide, and hesitation steps), which meant the group members may have needed to pay substantially higher attention to follow the musical beats which, in turn, may have resulted in slightly shorter step length/lower walking speed. This might not have been the case in the paced-walking group, which was exposed to a clear and fixed auditory tone. Nevertheless, walking while listening to music beats can increase enjoyment and promote contemplation among the sedentary population. This may be the first long-term longitudinal study on music-movement synchrony; to verify any significant effect of such walking, future studies should consider assessing exercise intensity and cognitive/attentional resource use (using the present study’s task paradigm) during music-synchronised walking [42].

Interestingly, our study investigated retention of the intervention’s effects at 12 weeks after the programme end. During these 12 weeks, all participants were asked to resume their usual/preferred lifestyles without performing supervised exercise. In general, only HDL-C and VO_2_max levels remained robust after the retention period. Interestingly, only the normal-walking group retained the step length improvement. A possible reason for this is the differences between the paced and music-synchronised walking groups in regard to the task requirements during the training (with tone/music presented) and the evaluation sessions (no tone/music presented); the participants may have “relied on” feedback from these tones/music beats to walk faster or with a longer step length. However, body mass and fat ratio also deteriorated to the pre-exercise level, indicating that these variables are relatively sensitive to exercise interventions. One plausible explanation for the loss of improvement following completion of the 24-week training is that, during the 24-week intervention, exercise participation was motivated by peer support, the supervised nature of the programme, and the incentives provided. The subsequent absence of such incentives and support during the retention period is likely to have decreased the participants’ motivation. This partly accords with the findings of a previous study [43], which showed that an incentive-based programme can induce positive effects on physical activity, sedentary behaviour, and physiological biomarkers. Another plausible explanation concerns the change in the outdoor climate from the training period (June to November) to the retention period (December to February). The average outdoor temperature in Beijing ranged from 40 °F to 77 °F during the training period, but fell to 26–32 °F during the retention period (https://weatherspark.com/y/131055/Average-Weather-in-Beijing-China-Year-Round). This may have influenced the participants’ motivation to engage in outdoor physical activity behaviour [44, 45]. Our findings provide indirect evidence that regular exercise habits should be maintained and encouraged in sedentary pre-older females. Future studies should consider including a group that is encouraged to continue the walking intervention indefinitely in order to identify whether such a walking intervention is sustainable.

This study has some limitations. First, while we aimed to control the walking intensity under different walking conditions, a dose-response experiment would be necessary to better illustrate the association between walking type and physiological effects. Moreover, different physiological parameters may have different adaptation times, and the implementation of wearable biosensor devices could prolong the monitoring of physiological changes. Second, there was a high dropout rate, which is also commonly found in studies involving sports participation; nevertheless, this may have contributed to attrition bias in the findings [46, 47]. Tiredness was the primary reason for participant withdrawal; however, motivational loss is often observed in individuals who do not habitually perform exercise [48]. In compliance with ethical requirements, the participants were free to discontinue the study at any point, regardless of our incentives, the presence of peer encouragement, and the supervisory protocol design. In addition, it has been reported that females aged over 46 years perceive social pressure to quit engaging in exercise; for example, some participants reported that they needed more time to arrange relocation for their families or to care for their children or grandchildren. Additionally, our requirement that the participants were habitually sedentary further increased the challenge of performing this study; despite this, we facilitated the presence of peer support for the participants by implementing a supervised programme and provided positive reinforcement via incentives. The supervised exercise approach increased the likelihood of compliance by helping the participants believe they had the behavioural capacity to complete the tasks, and also by providing them with encouragement to do so [49]. Nevertheless, the final sample size of 78, with four groups and measurement time points, returned a post-hoc statistical power of 0.88, assuming a small-to-medium effect size (*f* = 0.175). To encourage greater participation, future studies may consider incorporating mobile health (i.e. ‘mHealth’), exergames, or other metaverse approaches [50, 51]. It may also be useful to analyse the health belief/expectation model of sedentary behaviours in order to identify barriers to completion of exercise training programmes and to improve the setting of such programmes [52].

## Conclusions

Different walking-exercise programmes can improve some health parameters in sedentary pre-older females. Notably, all walking conditions tested in this study improved HDL-C, LDL-C, VO_2_max, VO_2_AT levels, and step length, with the benefits to HDL-C and VO_2_max being retained even after the participants returned to their usual lifestyle. Walking in time with an auditory tone/music appeared to induce additional health benefits when compared to normal walking. Further investigations should be conducted to identify the underlying mechanism for the effect combined music/tempo and movement have on physiological responses.

## Data Availability

The datasets used and/or analysed during the current study are available from the corresponding author upon reasonable request.

## Abbreviations

CO: Cardiac Output
EF: Ejection Fraction
FFQ: Food Frequency Questionnaires
FITT: Frequency, intensity, time, and type
HbA1c: Haemoglobin A1C
HDL-C: High-density Lipoprotein
IPAQ: International Physical Activity Questionnaire
LDL-C: Low-density Lipoprotein
MET: Metabolic Equivalent of Task
SV: Stroke Volume
VO_2_AT: Oxidative Capacity at Anaerobic Threshold
VO_2_max: Maximum Oxygen Consumption
